# Seaweeds in Food: Current Trends

**DOI:** 10.3390/plants12122287

**Published:** 2023-06-12

**Authors:** Cristian Rogel-Castillo, Monica Latorre-Castañeda, Camila Muñoz-Muñoz, Cristian Agurto-Muñoz

**Affiliations:** 1 Department of Food Science and Technology, School of Pharmacy, University of Concepcion, Barrio Universitario S/N, Concepción 4070386, Chile; cagurto@udec.cl; 2 Interdisciplinary Marine Biotechnology Group (GIBMAR), Biotechnology Center, University of Concepcion, Barrio Universitario S/N, Concepción 4070386, Chile; molatorre@udec.cl (M.L.-C.); camilamunozm@udec.cl (C.M.-M.)

**Keywords:** edible seaweed, bioactive compounds, nutritional composition, heavy metals, food safety

## Abstract

Edible seaweeds are an excellent source of macronutrients, micronutrients, and bioactive compounds, and they can be consumed raw or used as ingredients in food products. However, seaweeds may also bioaccumulate potentially hazardous compounds for human health and animals, namely, heavy metals. Hence, the purpose of this review is to analyze the recent trends of edible seaweeds research: (i) nutritional composition and bioactive compounds, (ii) the use and acceptability of seaweeds in foodstuffs, (iii) the bioaccumulation of heavy metals and microbial pathogens, and (iv) current trends in Chile for using seaweeds in food. In summary, while it is evident that seaweeds are consumed widely worldwide, more research is needed to characterize new types of edible seaweeds as well as their use as ingredients in the development of new food products. Additionally, more research is needed to maintain control of the presence of heavy metals to assure a safe product for consumers. Finally, the need to keep promoting the benefits of seaweed consumption is emphasized, adding value in the algae-based production chain, and promoting a social algal culture.

## 1. Introduction

Seaweeds, or macroalgae, refer to numerous species of multicellular photosynthetic organism. These can be classified into three major groups: brown algae (*Phaeophyta*), red algae (*Rhodophyta*), and green algae (*Chlorophyta*) [1]. Overall, seaweeds are distributed along shores and solid substrates, and, ecologically, seaweeds play an important role because they use sunlight to produce food through photosynthesis that can then be used by the marine food web [2].

The seaweed industry (both farming and from natural seaweed beds) experienced a significant almost threefold increase from 2000 to 2019. In general, seaweed production mainly comes from Asia, which accounts for almost 98% of the world’s production. In particular, China is ranked first in the world in terms of production followed by Indonesia. Other countries that contribute to seaweed production include South Korea, the Philippines, Japan, and North Korea. Moreover, most seaweed for industrial use is cultivated, while just a small amount comes from wild harvests. The main edible seaweeds are Japanese kelp (*Laminaria japonica*), Kombu (*Sacharinna* spp.), *Gracilaria* spp., Nori Nei (*Porphyra* spp.), *Eucheuma* seaweeds nei (*Eucheuma* spp.), Laver or Nori (*Porphyra tenera*), Wakame (*Undaria pinnatifida*), Elkhorn Sea moss (*Kappaphycus alvarezii*), Hijiko or Hiziki (*Sargassum fusiforme*), Umudggasari (*Gelidium amansii*), and Gamtae (*Eckonia cava*), among others [2,3]. Of these seaweed species, Chile is the main exporter of *Gracilaria* spp. and *Durvillaea antarctica*, which both come primarily from natural seaweed beds. Most of the raw seaweeds in Mexico, United States, Peru, and Canada come from natural seaweed beds also. The same occurs in France, Scotland, Spain, Ireland, and Norway where almost all seaweeds (e.g., Dulse, or *Palmaria palmata*) are harvested from natural seaweed beds [3]. However, there are approximately 200 companies producing macroalgae that are primarily located in France, Ireland, and Spain. Of the seaweed species cultivated, *Saccharina latissima* has the greatest production volume followed by *Alaria esculenta* and *Ulva* sp. [4]. It is noteworthy that around 80% of the seaweed in Africa (ca. 0.4% of the world’s production) comes from seaweed farming rather than wild harvesting, with *Eucheuma denticulatum* being the main seaweed cultivated. A similar trend can be observed in Oceania where almost all seaweeds come from cultivation [3]. For additional information on seaweed cultivation worldwide, the reader is suggested to read the papers published by Zhang, L. et al. [3] and Cai, J. et al. [5].

While seaweeds are currently used worldwide and are growing in usage, there is more potential. Based on current trends, it is estimated that the global population will reach ca. 11 billion by 2100, and additional food production will be needed for this growing population. Seaweeds could be an alternative food source for both humans and livestock. Thus, both seaweed farming and wild harvest, along with developing innovative new seaweed-based edible products, are viable alternatives for achieving economic development in emergent economies as well as sustainable development [6].

Edible seaweeds are consumed directly, for example, as an ingredient for sushi wraps or in salads and soups. Hence, based on their nutritional composition, they can be classified as “novel food” according to Regulation (EU) 2015/2283 [7]. In general, seaweeds contain macronutrients, including carbohydrates (dietary fiber), proteins (essential amino acids), and lipids (essential fatty acids, n-3 and n-6), and micronutrients, such as minerals and vitamins. These components have shown various health benefits, such as anti-inflammatory, anti-obesity, anticancer, antioxidant, and antibacterial [8]. In addition to macronutrients, several red seaweeds, such as *Gelidium*, *Gracilaria*, and *Neopyropia*, are used for agar and carrageenan production, and brown seaweeds are used for alginate production, which are commonly used in cosmetics, food additives, ingredients in functional food, and pharmaceutical products [1,9,10,11,12].

While the consumption of seaweed has a number of health benefits, seaweeds can also accumulate hazardous compounds, such as heavy metals and pathogenic microorganisms that could potentially adversely affect people to varying degrees. Hence, every country should establish standards and protocols of good practices for the handling, cultivation, harvesting, and processing of seaweeds to ensure their quality for human consumption. In this regard, for example, Chile has in the last decade developed a regional effort to promote the consumption of seaweeds. This has involved the implementation of several projects that focus on the dissemination, valuation, safety, and innovation in seaweed-based food.

Hence, the purpose of this review is to analyze the recent trends in the field of edible seaweeds on the following topics: (i) nutritional composition and bioactive compounds, (ii) the use and acceptability of seaweeds in foodstuffs, (iii) the bioaccumulation of heavy metals and microbial pathogens by edible seaweeds as a matter of food safety, and (iv) current trends in Chile for using seaweeds in food.

## 2. Nutritional Composition and Bioactive Compounds in Edible Seaweeds

Seaweed constitutes an important marine resource. Despite their use in the hydrocolloid (agar, carrageenan, alginate) industry and their highly nutritional composition and the presence of bioactive compounds, the development of innovative products, such as nutraceuticals or functional foods, has not been the main objective when using these resources [8,13,14]. Instead, research has focused on the chemical extraction and the characterization of bioactive compounds from different types of edible seaweeds. More information about the latest advances in extraction techniques can be seen in [15,16].

Bioactive compounds commonly found in seaweed include polysaccharides (e.g., sulfated, alginate, carrageenan, agar, dietary fiber, laminaran) [17,18,19], lipids and fatty acids (e.g., essential fatty acids), proteins and peptides [20,21], carotenoids and polyphenols [22,23,24], and minerals (such as iodine) and vitamins, and different methods are used to extract these compounds. For instance, bioactive compounds (protein, phlorotannins, and antioxidant) obtained from *Durvillaea potatorum* (Australian Bull Kelp) have been encapsulated using alginate and their release was evaluated in an in vitro gastrointestinal model [25]. In another study by Naveen et al., the researchers profiled the bioactive compounds obtained from the brown seaweed *Padina tetrastromatica*. As well as alginate, the authors reported the presence of luteolin, epigenin, glycolipid, α-linoleic acid, and fucoxanthin as well as a significant quantity of polyphenols, which have antioxidant and antidiabetic properties [26]. *Padina tetrastromatica* was also evaluated for its polysaccharide, fucoxanthin, and lipids content and their use as a functional ingredient for weight management. Overall, the results demonstrated that fucoxanthin, polysaccharide, and total lipids presented an anti-obese effect by reducing body weight in mice [27].

For the fatty acids (FA) obtained from seaweeds, the FA profiles of seaweeds are characterized by the presence of essential fatty acids n-3 and n-6 overall. For example, Rocha et al. evaluated the potential of harvested red seaweeds (*Asparagospis armata*, *Calliblepharis jubata*, *Chondracanthus teedei* var. *lusitanicus*, *Gracilaria gracilis*, and *Grateloupia turuturu*) and brown seaweeds (*Colpomenia peregrina*, *Sargassum muticum*, and *Undaria pinnatifida*) from Portugal as effective sources for essential FA [28]. In general, the results showed that *C. jubata* and *U. pinnatifida* had the highest nutraceutical potential. Moreover, Soares et al. determined the total lipid content and lipidic profile of seaweeds collected from the Portuguese North Coast. In general, it was found that the total lipids varied between species of seaweeds in the range 0.7 ± 0.1% (*Chondrus crispus*) and 3.8 ± 0.6% (*Ulva* spp.). For the fatty acid content, polyunsaturated fatty acids (PUFA) fluctuated between 0 and 35% with *Ulva* spp. exhibiting the highest amount; monounsaturated fatty acids (MUFA) varied between 19 and 67%; and saturated fatty acids (SFA) were prevalent in *C. crispus* (45–78%) and *Gracilaria* spp. (36–79%) [29]. Other authors obtained the fatty acid profile for *Sargassum miyabei* [30] as well as the fatty acid profile of seaweed from the Arabian Gulf region [31].

Other components obtained from seaweed are natural antioxidants, such as carotenoids. For instance, a carotenoid, namely fucoxanthin, was obtained from *Durvillaea incurvata* after drying the seaweed using an alternative drying process to protect the carotenoid. In addition, the nutritional content was evaluated and consisted of dietary fiber (56.9 g/100 g, dry weight), ash (20.9 g/100 g, dry weight), and protein (8.30 g/100 g, dry weight) [32]. The antioxidant activity was determined using 2,2-Diphenyl-1-picrylhydrazyl (DPPH) assay and Oxygen Radical Absorbance Capacity (ORAC) assay, and was dependent on the extract concentration. In the same way, the antioxidant activities of *Kappaphycus alvarezii*, *Kappaphycus striatus* [33], and *Padina gymnospora* [33,34] were also determined.

Other bioactive compounds that have attracted researchers are those with antimicrobial activity, especially against multidrug-resistant microorganisms and foodborne pathogens that can affect the shelf-life and quality of foods [35]. For example, an extract obtained from the Malaysian red seaweed, *Gracilaria edulis*, was evaluated for its potential antibacterial activity against six multi-resistant bacteria, namely *Klebsiella pneumoniae*, *Pseudomonas aeruginosa*, *Salmonella enterica*, *methicillin-resistant Staphylococcus aureus (MRSA)*, *Streptococcus pyogenes*, and *Bacillus subtilis*. In summary, the results showed that Gram-positive and -negative bacteria were inhibited by the extracts obtained from *Gracilaria edulis* [36].

Concerning seaweed as a protein source, it is recognized that seaweeds are a promising alternative protein source for global food sustainability [37]. Based on this, Ummat et al. evaluated the effect of processing conditions in the extraction of protein, free amino acids (FAA), and umami FAA from Irish brown seaweeds (*Ascophyllum nodosum* and *Fucus vesiculosus*). The results revealed that both seaweeds were good sources of protein and FAAs, and the potential use of these as flavoring agents in the food industry was established [38]. In addition, the in vitro digestibility of proteins extracted from Irish seaweeds *Palmaria palmata*, *Fucus serratus*, and *Alaria esculenta* demonstrated them to be a good source of essential amino acids, such as lysine, methionine, and tryptophan as well other important amino acids [39].

In Chile, Véliz et al. determined the chemical composition of eleven seaweeds: the green seaweed *Ulva* sp.; the brown seaweeds *Durvillaea incurvata*, *Lessonia spicata*, *Lessonia berteroana*, *Lesonia trabeculata*, and *Macrocystis pyrifera*; and the red seaweeds *Gracilaria chilensis*, *Chondracanthus chamissoi*, *Cryptonemia obovata*, *Sarcodiotecha gaudichaudii*, and *Acrosorium* sp. Overall, the results showed variability among the seaweeds. For instance, the ash content ranged from 18.5 ± 2.0% in *Ulva* sp. to 50.8% in *M. pyrifera*. Total lipids varied from 0.9 ± 0.4% in *C. chamissoi* to 3.9 ± 1.8% in *Ulva* sp. The protein content was between 6.5 ± 1.1% in *D. incurvata* and 18.9 ± 2.3% in *S. gaudichaudii.* Crude fiber varied from 2.5 ± 1.0% in *C. chamissoi* to 9.5 ± 3.6% in *L. spicata*. Regarding the contents of nitrogen-free extract, values were between 31.9 ± 12.0% in *M. pyrifera* and 61.2 ± 6.0% in *C. chamissoi*. In relation to mineral contents (Na, K, Ca, Mg, P, Fe, Mn, Cu, Zn, Mo, Se), in general, they were within the Recommended Daily Allowance (RDA) according to the Commission Directive 2008/100/EC [40].

## 3. Use and Acceptability of Seaweeds in Foodstuffs

Edible seaweeds have an established market in Asia and a growing market in Europe [41]. The seaweed market has increased in popularity in recent years due to new trends in functional foods as people become more aware of the foods in their diet, their health impact, and their origins. Generally, it is believed that edible seaweed can be less harmful to the environment and offer a wide variety of benefits by either supplementing diets as a food additive or being directly consumed [42]. For example, seaweed could be a substitute for meat, and this, according to the United Nations (UN), will increase by 75% by 2050. Seaweeds are a potential food substitute that could mitigate the excess consumption of animal protein, and thus address the environmental damage that meat production entails [43].

Seaweeds and algae-derived bioactive compounds play a major role in humans as a beneficial element, and edible seaweeds stand out worldwide. The Food and Agriculture Organization of the United Nations (FAO) considers edible seaweeds the ideal diet of the 21st century because of their high protein, low fat, low sugar, and low cholesterol content. Today, seaweed is used as much as vegetables, and in many Asian countries, seaweed is an important part of the human diet in fresh, dried, flake, and meal form [3].

Main challenges of this emerging market are the lack of awareness about the benefits of seaweed when incorporated into food, and the lack of regulations regarding seaweed cultivation and harvesting from natural seaweed beds for the mass production of seaweed-based products. For example, recent studies have shown “food neophobia” as a response to the consumption of foods containing a high percentage of seaweed in their composition [44,45]. Another study examined the potential market of seaweed-based products on the US market. The results suggested that there is a potential market for seaweed food products among US consumers since 35% of participants chose to purchase at least one seaweed product. Additionally, the price seemed to be an important factor in the consumer’s decision about trying seaweed food products [46]. A similar study was performed in terms of the perception of people in the United Kingdom towards seaweed and seaweed-based products. Some factors that influence a consumer’s seaweed consumption include sensory aspects such as taste, smell, texture, knowledge about health benefits and risks, affordability, and availability. For instance, 46% of the participants were not aware of the health benefits of seaweeds [47]. Traditionally, countries in Asia have higher annual per capita consumption rates than those in Europe or America [5]. However, these percentages are increasing [41]. Efforts to apply seaweeds and generate new products are focused on familiarizing consumers with the nutritional benefits and sustainable cultivation of seaweeds in addition to accepting their organoleptic properties, such as smell, taste, and texture. While processing methods, as well as storage conditions, can influence flavor, it has been predicted and demonstrated that the general acceptability of seaweed in the human diet would be increased by incorporating them into foods. For example, seaweed flavor is more accepted when mixed in low doses in traditional foods, such as cookies or chocolate. Other proposed strategies include improving the sensory characteristics during seaweed processing, such as the development of mutant seaweed with low chlorophyll, but the acceptability depends on the food product they are used in. Algal food design is essential for market integration because seaweed is generally not part of Western cuisine or diet. Additionally, sometimes it is perceived to have an unpleasant or fishy taste and odor. To integrate seaweed more into Western cuisine, new seaweed food designs are required, and they include strategies to promote and establish the massification of seaweed products in markets [41]. Some countries worldwide are progressing faster than others in this regard. For example, in the case of Chile, a series of research and innovation projects have promoted the use of seaweed for the development of new food products, food supplements, and recipes, for which the seaweeds being used include *Ulva* sp. (green seaweed), *Durvillaea incurvata* (brown seaweeds), and *Chondracanthus chamissoi* (red seaweeds) (see Section 5).

In general, approximately 70% of seaweeds are used for food consumption (predominantly), while the remaining approximately 30% are used as fertilizer and feed, among other uses [48]. Edible seaweed can be consumed on its own or as an ingredient in prepared foods. These forms for consumption include fresh, fermented, dried, frozen, whole, ground into flakes, granules, and powders. Examples of food products with seaweed ingredients are cookies, pasta, bread, and beverages [41].

In addition to food consumption, seaweeds can be used as food additives, which are substances that are added to foods to maintain or improve their safety, freshness, flavor, texture, or appearance. According to the World Health Organization (WHO) and FAO, food additives include flavorings, enzyme preparations, and other additives that are used for a variety of reasons, such as preserving, coloring, or sweetening [42]. Additives are essential to preserve the safety of processed foods and to maintain them in optimal condition. They are used for their well-defined technological need and purpose, such as preserving the nutritional quality of foods or improving their stability. Food additives can be added during food preparation, packaging, transportation, or storage, and are an ingredient in the final product. Food additives that are preservatives can slow food spoilage caused by oxidation, molds, bacteria, and yeasts. In addition to maintaining food quality, they help prevent contamination that can lead to foodborne illnesses. In this sense, we can find potential in the use of seaweed powders and extracts against lipid oxidation in food and oxidative stress in target tissues [3].

Traditionally, the uses of seaweeds are based on the improvement of physicochemical or rheological properties, such as stabilizing agents, thickeners, gelling agents, flocculants, water retention capacity, and emulsifiers. It is known that seaweeds contain chemical compounds such as polysaccharide, i.e., carrageenan, alginate, and agar, which are used as hydrocolloids (phycocolloids) [48].

Polysaccharides within seaweeds contribute to their binding properties and to the improvement in rheological behavior, texture, and organoleptic and microbiological properties. Carrageenan, agar, and alginate are water-soluble polymers that can produce high-viscosity solutions when dissolved in water. These polysaccharides have been widely used as food ingredients because of their unique functional properties, such as gelling, stabilization, emulsification, and thickening. These properties have enabled the development of numerous food products. In addition, these compounds are “generally regarded as safe substances (GRAS)” for human consumption by the European Food Safety Authority (EFSA) (Parma, Italy) and the Food and Drug Administration (FDA, Silver Spring, MD, USA) [49].

In addition to being used for the properties specified in the previous paragraph, seaweeds have been studied for other uses in food products. For example, generally, the meat industry uses a wide range of ingredients with specific technological properties to improve the appearance, flavor, and texture of products, as well as their nutritional value. Seaweed with a low caloric content, high content of key nutrients, dietary fiber, and health-promoting compounds can be used to overcome some technological problems associated with these reformulated meat products through their fat- and water-binding properties. For instance, the performance of *Undaria* sp. as a functional ingredient in low-fat pork patties was evaluated. The results showed that the fatty acid profile was comprised mainly of a high proportion of saturated fatty acids (SFA) and monounsaturated fatty acids (MUFA) (48.8% and 38.2%, respectively), followed by polyunsaturated fatty acids (PUFA) (13%) with palmitic (C16:0; 36.9%) and oleic (C18:1n-9; 34.4%) acids in higher abundance. The addition of this seaweed to the patties did not affect the fatty acid profiles or the fat content of the product. Therefore, it could be a suitable method for developing meat products with improved characteristics and antioxidant properties [23].

In another study, two different types of red (*Porphyra umbilicalis* and *Palmaria palmata*) and brown (*Himanthalia elongata* and *Undaria pinnatifida*) edible seaweeds were evaluated as salt replacement in reformulated frankfurters. In particular, salt addition and pork fat content were reduced by 50% and 21%, respectively. The reformulated frankfurters containing seaweed had less ash, more moisture and protein, a darker color, and altered textural properties compared to the control; furthermore, they were less hard and chewy. The overall acceptability of reformulated frankfurters containing seaweed was strongly influenced by the type of seaweed added, with reformulated frankfurters containing *H. elongata* being the most acceptable. The addition of *U. pinnatifida*, *P. umbilicalis*, *P. palmata*, and especially *H. elongata* have the potential to improve nutritional quality mainly through salt reduction. The authors highlighted that the frankfurters reformulated with the inclusion of *H. elongata* were the most promising, although more work is required to optimize the formulation [50].

In a different study that also used frankfurters, seaweeds were utilized as a source of dietary fiber (SDF) to address the technological problems associated with phosphate-free emulsified meat products. The incorporation of SDF can be used as a potential alternative to phosphates. In addition, the quality defects of phosphate-free products as well as inhibition of lipid oxidation were improved by using SDF. The best optimal phosphate substitution effect was observed with 1.00% SDF. In addition, hydrogen bonds and hydrophobic interactions were the main molecular strength in phosphate-free frankfurters added with SDF [51].

Other food additives commonly used in foods are colorants, and seaweeds are becoming a novel source of natural lipophilic pigments, which are valuable food ingredients. A study evaluated the content of lipophilic pigments in 20 brown and 4 red seaweeds from the coast of northern Spain and focused on the content of chlorophylls and fucoxanthin (carotenoid) with these seaweeds. The results showed that the highest content of the lipophilic pigment was among members of the order Fucales and *Undaria pinnatifida* [52], and these could be used as natural food colorants.

## 4. Bioaccumulation of Heavy Metals and Microbial Pathogens by Edible Seaweeds as a Matter of Food Safety

As mentioned in the previous section, seaweeds contain biologically active compounds, such as essential amino acids, essential fatty acids, and minerals (K, Na, Ca, Mg, Fe, Zn, I, Se, among others). Moreover, they comprise a variety of polysaccharides and proteins that contain a wide variety of chemical groups, such as sulfate, anionic carboxyl, and phosphate groups. However, these chemical groups mean that seaweeds have a high affinity for other components, e.g., heavy metals. Consequently, seaweeds may accumulate and become a source of arsenic (As), cadmium (Cd), lead (Pb), mercury (Hg), and other compounds [53]. Therefore, this raises the following questions: (i) does the consumption of seaweed foods and seaweed-based products contribute positively or adversely to human health, and (ii) should their consumption be encouraged or limited?

Heavy metals present several health risks to humans and animals, even at low concentrations. Their toxicity depends on the dose, the route of entry, and the duration of exposure, i.e., acute or chronic [54]. The occurrence of heavy metals in seaweeds depends on environmental factors, such as salinity, temperature, pH, light, and oxygen, and the type of seaweed [2,53]. Furthermore, heavy metal occurrence depends on the availability of metals coming from natural geological sources in addition to urbanization and industrial activities, which can all contribute to the contamination of aquatic systems. Consequently, heavy metals from anthropogenic activities, such as petrochemical processing or municipal waste, may reach areas where seaweeds are cultivated or grow naturally. In addition, the accumulation of heavy metals in seaweed can come from natural sources, including but not limited to volcanic activities. For example, Chile has metal exposure due to natural geological sources. Because of seaweed’s ability to absorb heavy metals, it is often used for biomonitoring of natural and anthropogenic contaminants to assess the health of marine ecosystems [1,12].

To evaluate the presence of heavy metals in seaweeds, several studies have been conducted and are detailed in the paragraphs below. For instance, Peng et al. compared and quantified the presence of 13 elements, including Cr, Cd, As, Ag, and Pb, in Phaeophyta species (*Sargassum polycystum*, *Sargassum oligocystum*, *Sargassum thunbergii*, *Padina crassa*, and *Turbinaria ornate*), Rhodophyta species (*Asparagopsis taxiformis*, *Gracilaria eucheumatoides*, *Gracilaria tenuistipitata*, and *Chnoospora implexa*), and Chlorophyta species (*Caulerpa lentillifera* and *Caulerpa racemosa*). This research included determining their heavy metal binding ability through the species-specific bioaccumulation factors (BAF) and performing a health risk assessment (HRA) for both children and adults. The seaweeds were collected from Hainan Island, South China Sea. The results showed that the red algae mostly contained V, Se, Mn, Ni, and Ag; the brown algae primarily had Cr, Co, Cu, Cd, As, and Fe; and the green algae mostly possessed Zn and Pb. *Padina crassa*, *Sargassum thunbergii*, *Caulerpa racemosa*, and *Asparagopsis taxiformis* had similar metal bioaccumulation behavior. With regards to the HRA that was calculated by estimating the Hazard Index (HI), the results showed that adults had an overall HI less than 1. On the other hand, the HI was greater than 1 for four seaweeds, *Sargassum oligocystum*, *Turbinaria ornate*, *Sargassum polycystum* and *Sargassum thunbergii*, when the HRA was performed considering seaweed consumption by children. In general, an HI above 1 suggests increased probability of a toxicological response. Therefore, in this case, the consumption of these seaweeds by children should be controlled. At the conclusion of the research, it was determined that both the bioaccumulation of heavy metals and the health risk associated with them vary significantly and are complex among different species [55].

In a different study performed by Huang, Bi et al., total arsenic and arsenic speciation was determined in 20 edible seaweeds, including *Saccharina japonica*, *Undaria pinnatifida*, *Neopyropia* spp., *Gracilaria* spp., and *Sargassum fusiforme*. All species were collected from six provinces along the Chinese coastline, and the influence of geographical location on arsenic concentration and speciation in cultivated *Saccharina japonica*, *Sargassum fusiforme*, *Sargassum horneri*, and *Undaria pinnatifida* in temperate zones versus subtropical zones was investigated. Additionally, the absorption of inorganic arsenic (iAs) was estimated in all the analyzed seaweed species to assess the safety of using seaweed as a food ingredient and additive. Overall, the total arsenic concentration fluctuated between 15.3 and 150 mg/kg for brown algae, 2.2 and 39 mg/kg for red algae, and 1.5 and 28.3 mg/kg for green algae. As for iAs concentrations, in general, they were below the maximum limits (0.3 mg iAs/kg) according to the National Food Safety Standard of Pollutants in China, and this limit is applied to seaweeds when they are used as additives for infant food. On the other hand, the results reported elevated iAs concentrations in four *Sargassum* spp. where the iAs ranged from 15.1 to 83.7 mg/kg. The authors indicated that the estimated daily intake (EDI) of iAs due to seaweed consumption was below the EFSA CONTAM Panel benchmark dose lower confidence limit (0.3 μg/kg bw/day), but it was higher for all *Sargassum* spp. Regarding the impact of geographical location on arsenic concentration, no significant differences were observed when comparing total As and iAs concentrations. In conclusion, based on the iAs concentration found, *Sargassum* spp. may present a health risk and its consumption should be reduced. In addition, it is important to have detailed and accurate information on arsenic speciation in seaweeds. This information, then, can be used to estimate dietary exposure in humans [10].

In a study conducted in Korea, samples belonging to 11 different types of seaweed from the major coastal cities were evaluated for Cd and Pb concentrations in addition to As species. The seaweeds considered were Laver (*Pyropia* sp.), Green Laver (*Enteromorpha* sp.), Sea Mustard (*Undaria pinnatifida*), Sea Tangle (*Saccharina japonica*), and Gulfweed or Hijiki (*Sargassum* spp.). The results revealed that the concentrations for Cd (range: 0.023–0.232 mg/kg fresh weight (fw)) and Pb (range: 0.025–0.222 mg/kg fw) were below the maximum limit in most international regulations for edible seaweeds. For As, the authors reported that the amount of total As was high (range: 1.020–20.525 mg/kg fw). When comparing the different seaweed groups, the *Sargassum* spp. had the highest level of inorganic As (sum of arsenate (As (V)) and arsenite (As (III))) ranging from 5.198 to 16.867 mg/kg fw, representing almost 69–85% of the total As concentration, while other species, such as *Pyropia* sp., *Enteromorpha* sp., *Undaria* sp., and *Saccharina* sp., primarily had non-toxic organic As (i.e., arsenosugars). While the As is high for *Sargassum* seaweeds, most of these are considered inedible apart from *Sargassum fusiforme* (Hijiki), which is considered an edible seaweed. The authors mention that according to statistical data from Korean and Japanese diets, the daily intake of *S. fusiforme* is low and therefore considered safe when consumed at the recommended daily intake. Despite the results, the authors recognized that the number of samples considered in the study were not enough to perform an evaluation of food safety. In conclusion, additional studies are needed to evaluate the risk of inorganic As in seaweeds using standardized methods and statistical relationships studies [56].

A study in Bangladesh analyzed a total of 13 elements (i.e., Pb, Cd, Fe, Mn, Co, Ni, Cu, Zn, Mg, Ag, As, Hg, and Se) from samples collected in 20 seaweed farms from the southeast coastal area of Bangladesh. The seaweeds were selected based on their commercial importance: *Caulerpa racemosa*, *Enteromorpha* sp., *Hypnea* sp., *Sargassum* sp., and *Gelidium* sp. After analysis, the results were as follows for the heavy metals: average Pb concentration 3.44 mg/kg, average Cd concentration 0.54 mg/kg, and average Hg concentration 0.13 mg/kg. In general, the concentrations were observed to be below the threshold level for human consumptions. Although several types of heavy metals were discovered in the seaweed samples, their concentrations were below the risk level for human health. Therefore, the authors emphasize that the seaweeds considered in this study are safe for human consumption, suggesting that these seaweeds can be considered for industrial uses and for exportation to international markets. While more studies and data are still needed regarding the presence of heavy metals in seaweeds, the authors highlighted that this was the first study to determine the presence of heavy metals in seaweeds from the coast of Bangladesh [2].

In research carried out in Malaysia, the concentrations of 17 elements, including Pb, Cd, and As, from locally resourced seaweed (three locations) were measured in addition to the assessment of potential noncarcinogenic and carcinogenic risks. Overall, low concentrations of potentially toxic elements, specifically Cd, Pb, and As, were found with overall mean values of 1.63 µg/g, 7.69 µg/g, and 4.40 µg/g, respectively. It should be noted that there was variation in the concentrations between the three locations. In general, most of the metals’ daily estimated intakes were within the level recommended by international organizations; however, the authors indicated that the estimated Hazard Index (HI), which represents noncarcinogenic (chemical) risk due to exposure from multiple metals, was greater than 1.0 (4.38). In this case, an HI greater than 1.0 indicates a probable health risk occurring from long-term consumption of the seaweeds assessed in the study. In the authors’ conclusions, they mentioned the need for detailed investigation of metal levels in the seaweeds collected from the selected locations, as well as the use of techniques to remove heavy metals, and that although the presence of heavy metals in algae should be scrutinized, they nonetheless remain a good source of macro and micronutrients with great health benefits for consumers [1].

As discussed so far in this review, seaweeds’ consumption is the basis of the diet of many Eastern Asian countries; however, in the last decade or so, seaweeds have become more popular in Western diets as exemplified in the number of studies conducted.

Research conducted by Babaahmadifooladia et al. performed screening for inorganic contaminants, including As, Cd, Hg, and Pb, in raw seaweeds, dried seaweeds, and food containing a fraction of seaweed for Belgian consumers. This information in conjunction with food consumption data was used in chronic probabilistic exposure calculations and risk characterization to evaluate the toxicological risk in the selected products. The exposure values were corrected by the bioaccessible fraction, which corresponds to the fraction that may be released during digestion, of the different metals to avoid overestimation in the reported exposures. The bioaccessible fraction was obtained from the average bioaccessibility (expressed as %) for dried Nori as the example since Nori was representative of dried seaweeds in the study. Overall, a decrease in the exposure values, based on the bioaccessible fraction, was obtained: As 56.00% (for average bioaccessibility), Pb 31.94%, Cd 27.48%, Ni 20.53%, and Hg 8.19%. When considering the corrected values, the authors indicated that no element exceeded the toxicological reference values. When comparing pure seaweeds and composite foodstuffs, it was found that pure seaweeds showed more instances of the approximation or exceeding of toxicological limits than composite foodstuffs because composite foodstuffs only contain a percentage of seaweed. Moreover, the authors highlight that due to increased consumption of seaweed and seaweed-based products as innovative foods, this may result in potential health issues. Therefore, the authors indicated that, based on the results, importance should be given to foods that are frequently consumed in larger amounts and especially those that contain higher concentrations of the elements considered in the present study [57].

Panebianco et al. analyzed the heavy- and semi-metals (namely, As, Cd, Cr, Hg, Pb, Tl) content in seaweeds in 26 samples obtained from ethnic food stores situated in southern Italy. In general, the samples were Konbu, Wakame, Arame, and Nori. Most of the samples did not report the scientific name of the seaweed in the packaging and their origin was mainly from China and Korea. Overall, the heavy- and semi-metal results indicated that As was most prevalent in the seaweeds (average = 8.19 ± 6.62 mg/kg), followed by Cd (0.38 ± 0.25 mg/kg), and Pb (0.12 ± 0.10 mg/kg), and very low concentrations of Cr, Hg, and Tl were found. For instance, the European Commission (EC) Regulation No. 1881/2006 and amendments define a maximum concentration of 3 mg/kg (wet weight) for Cd [58]. The levels of Cd in the samples were below this limit. Furthermore, when considering the maximum level established for Cd in France by the Center d’Etude et de Valorization des Algues (CEVA), 0.5 mg/kg dry weight, just five samples (19.2%) were above this limit. Further assessments of the data with chemometric analysis showed some trends between accumulations in product type (e.g., higher levels of As in Kombu seaweed) and geographic origin (e.g., similar levels in seaweeds from China and Korea). In summary, the study demonstrated that the heavy- and semi-metals’ content in commercialized seaweeds in southern Italy should not be of concern for consumers. Nonetheless, the authors emphasized that constant monitoring is still necessary to create a database on the presence of heavy- and semi-metals in seaweed foodstuffs found in southern Italy, and that the data obtained could be used for the risk assessment process [59].

In another study conducted in Italy by Filipini et al., the main objective was to analyze 20 heavy metals in addition to arsenic (total and inorganic fraction) in seaweeds, and health risk assessments were performed also. A total of 72 samples from 8 genera were purchased from a combination of large-scale retailers, small ethnic stores, and supermarkets. The sample distribution was brown seaweed (*Himanthalia n* = 8, *Saccharina n* = 15, *Undaria n* = 10, *Ascophyllum n* = 1, and *Laminaria n* = 1), red seaweed (*Porphyra n* = 13 and *Palmaria n* = 9), green seaweed (*Ulva n* = 10), and mixed seaweed (*n* = 5) with 19 samples originating from China and Korea and 53 samples originating from France, Spain, Germany, and Belarus. After analysis, the Al, Cd, Pb, As, and Hg concentrations for the different genera of seaweeds were obtained. Al had a concentration of 165.39 mg/kg for mixed algae (the highest) with 33.15 mg/kg for *Laminaria*, 29.11 mg/kg Al for *Ascophyllum*, and 0.71 mg/kg for *Himanthalia*. For Cd, *Porphyra* showed the highest concentration with 1.56 mg/kg. For Pb, the highest concentration was observed in mixed algae with 0.56 mg/kg, followed by 0.17 mg/kg for *Porphyra*, 0.17 mg/kg for *Saccharina*, and 0.16 mg/kg for Ulva. For total and inorganic contents of As, a higher concentration of total As was found in phylum Phaeophyta. Of the samples analyzed, 2 samples exceeded French and USA limits (3 mg/kg) for inorganic, and 15 samples exceeded the limits for the Australia New Zealand Food Standard Code (1 mg/kg iAs). From the Pb, Cd, Al, and As concentrations found in the samples, health risk assessments (HRA) were calculated for children and adults using the Hazard Quotient (HQ) and the Hazard Index (HI). Since at the time of the study there was no information about seaweed consumption in Europe, the authors used the United State Environmental Protection Agency (USEPA) guidelines. The results demonstrated that for brown, red, and green seaweed, the HI was less than 1.0, meaning that these two groups did not represent a health risk. The only exception was Al with an HI = 1.09 for children. In summary, the results positively support the importance of monitoring the occurrence of toxic heavy metals in commercial products for the safety of consumer health [12].

In a study performed in the US market that analyzed 26 different elements, including toxic elements (As, Cd, Pb, and Hg), 46 edible seaweed samples were purchased from grocery stores and online retailers. The samples were then grouped into 13 subgroups/species belonging to brown (*n* = 28), red (*n* = 16), and green (*n* = 2) seaweeds. The results showed that concentration values differed for the toxic elements and across the seaweed groups. For example, total As was higher in brown seaweeds than red and green seaweeds. The concentration ranged from 20.4 ± 10 mg/kg for Wakame stems to 93.2 ± 10 mg/kg for Hijiki (brown seaweed), while the concentration ranged from 3.05 mg/kg for Sea moss mix to 30.5 ± 1 mg/kg for Laver range (red seaweed) and 6.15 ± 0.6 mg/kg in Sea lettuce (green seaweed). The highest levels of As were observed in Hijiki (93.2 ± 10 mg/kg) and Kombu (67.4 ± 10 mg/kg). For Cd, in general, levels were variable with the following concentrations: 0.272–1.85 mg/kg for brown seaweed; 0.258–2.73 mg/kg for red seaweed; and 0.273 mg/kg for green seaweed. Pb average concentration was lower than As and Cd, and Hg levels were the lowest of all the elements considered in the present study. The authors underlined that their study supports the results from prior investigations that for most of the elements, the taxonomic species is one of the main factors in seaweed elemental accumulation [11].

In the case of South America, research into the heavy metal content in edible seaweeds is still nascent with not many studies analyzing this yet. Thus, Veliz et al. analyzed the presence of total As, Cd, Hg, Pb, Ni, Cr, and Al in eleven Chilean seaweeds, including the green seaweed *Ulva* sp.; the brown seaweeds *Durvillaea incurvata*, *Lessonia spicata*, *Lessonia berteroana*, *Lesonia trabeculata*, and *Macrocystis pyrifera*; and the red seaweeds *Gracilaria chilensis*, *Chondracanthus chamissoi*, *Cryptonemia obovata*, *Sarcodiotecha gaudichaudii*, and *Acrosorium* sp. The results showed that total As ranged from 4.3 ± 1.4 mg kg^−1^ in *Ulva* sp. to 35.6 ± 6.9 mg kg^−1^ in *L. spicata* with higher values being detected in brown seaweeds than green and red seaweeds. In the case of Hg and Cd, the Hg content was between 0.009 ± 0.001 mg kg^−1^ in *C. chamissoi* and 0.026 ± 0.008 mg kg^−1^ in *M. pyrifera*, while for Cd, the values ranged from 0.11 ± 0.04 mg kg^−1^ in *G. chilensis* to 6.2 ± 1.1 mg kg^−1^ in *L. berteroana*. With the results of concentrations, the study also performed a Health Risk Assessment using the Targeted Hazard Quotient (TQH) and the Hazard Index (HI) for each element. All the values were <1.0 for the TQH, which means that the exposure dose was lower than the Recommended Reference Dose (*RfD*) established by the USEPA, and HI was <1.0, indicating a minimal health risk due to the consumption of the selected seaweed. However, the authors noted that an HI > 1.0 was observed in *M. pyrifera*, an HI = 0.91 for *L. berteroana*, and an HI = 0.97 for *L. spicata*. Hence, these seaweeds should be monitored for heavy metals if they are used in food products. In summary, the contents of As, Hg, and Pb were below the limits set for food and feed ingredients with the Health Risk Assessment results supporting this [40]. Despite the limited research, the Chilean Health Ministry is developing risk studies on heavy metals in marine food resources that includes edible algae and their by-products, and several projects in Chile are focused on reducing the risk of the accumulation of heavy metals and the presence of pathogenic microorganisms in edible seaweeds. These projects have focused on directly and indirectly promoting (i) seaweed cultivation at sea in areas away from anthropogenic activities, (ii) seaweed cultivation on land, (iii) proper management of natural seaweed beds, and (iv) the implementation of good manufacturing practices in the processing of algal biomass. For more details, refer to Section 5.

As the research studies above demonstrate, heavy metals can be present in edible seaweed; however, they are not the only health risk when consuming seaweed. Considering that some seaweed can be consumed raw, food poisoning can be a concern, especially with foodborne pathogens. Seaweed can be contaminated during harvesting or handling. Because of these factors, microbiological food spoilage has also been a topic of investigation in seaweeds, as demonstrated in the research below.

Martelli et al. explored the microbial populations found in 14 commercial ready-to-eat (RTE) dehydrated seaweed, focusing on *Bacillus cereus* and *Listeria monocytogenes*. In addition, the authors evaluated if *B. cereus* could grow during refrigerated storage using the microbiological challenge test in commercial *Undaria pinnatifida* and *Palmaria palmata* RTE foods. The results showed the detection of marine bacteria *Listeria* spp. and *B. cereus* in addition to coliforms in the RTE dehydrated seaweed-based food products. The authors expressed that the presence of *B. cereus* in the samples pose food safety concerns and could suggest possible risks for human health when consuming RTE foodstuff. Regarding *B. cereus* growing during refrigerated storage, it was found that *B. cereus* did not proliferate. However, the authors emphasized that this finding did not mean that microbiological exposures could not happen. In conclusion, additional research is necessary to assess the risk associated between microbial presence and different consumption habits of algae [60].

In another study, seaweed samples of *Alaria esculenta* and *Saccharina latissimi* obtained from Scotland were evaluated for their microbiological and nutritional quality over a period of two years (2019–2020). In particular, during storage, microbiological analyses focused on the enumeration of Total Viable Counts (TVC); *Pseudomonas* spp., *Enterobacteriaceae*, and *Bacillus* spp.; and included yeasts and molds. In addition, the presence of human pathogenic bacteria was also examined. In general, the results revealed that initial populations of TVC were different depending on the year of harvest. The microbiological analyses showed the presence of *Psychrobacter*, *Cobetia*, and *Pseudomonas* species in *A. esculenta*; and *Psychrobacter* spp. and *Micrococcus* spp. in *S. latissima*. In addition, the authors determined that the optimal drying temperature was 50 °C for microbiological and nutritional quality, and these results underline the importance of drying and handling practices for microbiological quality. Therefore, these conditions need to be considered when evaluating seaweed products, especially since seaweed-based products can be considered very perishable foodstuffs because of their nutritional composition. In their conclusions, the authors stressed that producers and retailers should be aware of processing conditions and even consider further processing, such as freezing or any other method of conservation, to reduce potential health risks from microorganisms [61].

As mentioned so far, seaweeds are a source of essential nutrients, but at the same time, they can be a source of detrimental elements or microorganisms that can adversely affect human health. Therefore, the next question arises: what can be done to ensure the safe usage of seaweed? Up until this point, several research studies have investigated ways to decrease contaminants in edible seaweed. For example, *Sargassum fusiforme*, an edible brown seaweed known to bioconcentrate arsenic (As), was used as a model to evaluate the effectiveness of several treatment methods to remove As: hot water, acid treatment, and fermentation. The results from these treatments demonstrated that As content was most reduced by hot water, specifically from 76.18 mg/kg to 1.64 mg/kg using hot water at 60 °C for 120 min. The second most effective treatment was 0.4% citric acid. *Lactobacillus rhamnosus* fermentation was the least effective. At the same time, this method decreased some beneficial elements, such as Na, K, Fe, Cu, Zn, Ca, Mn, and Mg, by approximately 11–62%. Additionally, the changes in 17 organic acids (OA) and 25 amino acids (AA) were measured. The results demonstrated that *Lactobacillus rhamnosus* utilized some OA (e.g., 2-hydroxybutyric acid) and synthetized others (e.g., 4-hydroxyphenylacetic acid). While *L. rhamnosus* did not use AAs, it synthesized other AAs during fermentation [9]. In another study, the influence of hydrothermal processing, specifically boiling water, on several micronutrients, i.e., I, Na, K, Se, and total arsenic (tAs) concentrations for *S. latissima*, *L. digitata*, *U. pinnatifida*, and *C. crispus* was evaluated. The results demonstrated that, particularly for I, tAs, and Se, the pattern for leachable fractions was as follows for the species examined: *L. digitata* ≥ *S. latissima > C. crispus > U. pinnatifida*; for Na: *S. latissima* > *L. digitata* > *C. crispus* > *U. pinnatifida*; and for K: *U. pinnatifida* > *L. digitata* > *S. latissima* > *C. crispus*. The authors concluded that seaweed processing and handling could be an approach to guarantee the safety of eating seaweeds while simultaneously preserving the health-balanced essential nutrients for consumers. Therefore, individual methods could assure the best seaweed nutritional composition without the negative effects of highly toxic elements. For future research, the authors emphasized that the next steps would be to evaluate the effect of hydrothermal processing on the bioavailability of nutrients. In addition, it should be noted that the results observed in this study could be used by consumers for in-home processing [62].

Based on the information reviewed thus far, we can answer the questions mentioned at the beginning of Section 4 regarding their positivity and the adverse effects on human health, and whether their consumption should be encouraged or limited. If we consider the benefits against the risk, it has been proved that the consumption of seaweeds and their products are, by far, beneficial for human health due to their bioactive compounds, such as fatty acids, fiber, minerals, and vitamins. In this regard, seaweed consumption should be encouraged, but the presence of potentially hazardous compounds should also be considered, such as inorganic arsenic (iAs). Current research has demonstrated that, in general, the levels of these compounds are below the maximum allowed concentration. Nevertheless, more research is needed in terms of risk assessment analysis and seaweed consumption. It should also be noted that different seaweeds species have different affinities for the hazardous compounds. For example, brown seaweed seems to concentrate more iAs than green or red seaweeds. Therefore, the consumption of brown seaweed should be controlled. However, if brown seaweed consumption cannot be controlled, preference should be given to seaweeds harvested from areas without anthropogenic intervention, such as away from industrial zones. Moreover, current research has shown that consumers could decrease the content of the toxic compounds by performing simple treatments, such as washing or boiling, for the seaweed before consumption. At the same time, these treatments could remove foodborne pathogens.

## 5. Current Trends in the Use of Seaweeds in Food: The Case of Chile

In Chile, there is no culture regarding the consumption of seaweed-based food despite the great availability of edible seaweeds on Chile’s coasts. While no official information is present, a non-official study conducted in 2019 by the “Undersecretariat of Fisheries and Aquaculture” (SUBPESCA) established that the Chilean population consumes approximately 15 kg of seafood products per year, of which only 0.19 kg corresponded to seaweed [63]. Of all potentially edible seaweeds in Chile, only two species are commonly consumed: “Cochayuyo” (*Durvillaea antarctica*) and “Luche” (*Porphyra* sp.). The low consumption rate can be attributed to several factors: lack of knowledge of the nutritional properties of seaweeds, negative sensory characteristics (smell, taste, and flavor), and the lack of food industry products offered where the main ingredient is seaweed. The study below exemplifies the economic and marketing aspects, among others, of increasing seaweed consumption in Chile.

In the project called, “Incorporación de la Industria Alimentaria de Consumo Humano Directo como Fuente de Agregación de Valor para las Macroalgas Nacionales” (Incorporation of the Food Industry for Direct Human Consumption as a Source of Value Addition for National Seaweed) [64], the main objective was to analyze the economical aspect of introducing Chilean seaweeds to the national and international market. The project confirmed that in Chile, as in many other countries, seaweed consumption is not part of the food culture. For this reason, there are not many incentives for the increased usage or cultivation of seaweeds in Chile except for those already used for direct human consumption in the country, i.e., *Durvillaea antarctica*, *Chondracanthus chamissoi*, *Porphyra* sp., *Callophyllis variegata*, and *Ulva* spp. For the seaweeds that are used, they are primarily collected using artisanal fishing collection methods, and they are generally used within the country for direct human consumption using traditional cultural methods. Essentially, this means the seaweeds are extracted manually and then are usually dried using traditional cultural methods (e.g., sundried) before being used in traditional food preparation. This is dissimilar to other countries that use mechanical means for harvesting seaweeds, such as using specialized boats and ships. Currently, Chile heavily regulates the extraction, cultivation, processing, and commercialization of marine resources; however, despite the regulations in place, there are still inadequacies in all phases of seaweed production. With increased growth projections for the production and commercialization of edible seaweed food products, the industry will need to integrate sustainable practices into their seaweed supplies, which could result in an increase in seaweed cultivation. In addition to this general finding, the project had other conclusions for expanding Chilean seaweeds into the nation and international markets. In general, more research and evaluation are required from preliminary harvesting phases to regulations. For example, more research is required to support basic scientific development for innovation and commercialization, and further evaluation of the existing Chilean seaweed resources and species will also make it possible to estimate sustainable utilization levels for each species according to biological, ecological, and environmental impacts. The Chilean seaweed industry must also solve the problems of raw material supply, which includes determining sustainable supplies that would include both the cultivation and management of natural seaweed beds. The supply issue would also include solving the legal difficulties for access, such as the permits that would be required. The Chilean coastal economy would also need help to improve productive development for seaweed harvesting, and training in species identification, and extractive and processing methods needs to be promoted for extractors. This includes the implementation of mechanized processes for harvesting and primary processing. Even with these in place, the development of high value-added products to utilize the seaweeds harvested would need to be performed. Finally, regulations would need to be adapted and differentiated according to the different productive activities [64].

The above project identified problems for expanding the utilization of Chilean seaweeds nationally and internationally. This included the lack of seaweed usage in the food cultures, such as in Chile. However, in the last decade, a series of local projects have been developed to promote the consumption of edible seaweed in Chile itself.

In 2012 a project called “Uso y Aplicaciones de Algas Marinas Chilenas para Consumo Humano” (Use and Applications of Chilean Seaweed for Human Consumption) [65] was developed with the objective of introducing and increasing the consumption of seaweed in the diet of the population and generating new food products based on Chilean seaweeds from the Los Lagos Region. The seaweeds *Callophyllis variegata* and *Chondracanthus chamissoi*, as well as *Durvillaea antarctica* and *Porphyra* sp., were selected for promotion in the initiative. The initiative included the selection of natural seaweed beds, harvesting, and nutritional characterization of the described seaweeds as well as the dissemination of new food product preparation ideas for direct consumption to consumers. For example, during the project, training and gastronomy workshops were held and seaweed-containing products were developed, such as energy bars, nachos, snacks, and more. One of the end products of the project was the recipe book, “Cocinando con algas de la Huerta del Mar” (Cooking with seaweed from the Huerta del Mar) [66] that contains different sweet and savory recipes. The project also examined the potential of training artisanal fishermen on how to generate added value to their products (e.g., fish, shellfish, and seaweed) while simultaneously improving the quality. This training could thus increase the fishermen’s income and quality of life.

In addition to the project promoting seaweeds from Los Lagos Region, there have been two different projects in the Coquimbo Region. In the project called “Transferencia de Tecnología para el desarrollo de Cocina Patrimonial y de Autor en base a Algas y Productos Marinos de la Región de Coquimbo” (Technology Transfer for the Development of Heritage and Signature Cuisine based on Seaweed and Marine Products from the Coquimbo Region), the objective was to disseminate and transfer knowledge of techniques and best practices to restaurants that would allow them to innovate in their gastronomic offerings using seaweed and marine products from the Coquimbo Region. Another objective was to diversify the menu of the restaurants through the development of heritage cuisine in addition to the inclusion of new recipes and signature preparations based on seaweed and marine products The participating restaurants in this project were identified with a seal that read “Aquí comemos algas” (Here we eat seaweed). Cooking workshops were used for the restaurants to teach techniques and best cooking practices for the preparation of seaweed, and several restaurants included different seaweed preparations in their menus for their customers [66].

The other project in the Coquimbo Region, called, “Nodo Comercialización de Productos de Origen Algal como Alimento Funcional en la Región de Coquimbo” (Node for marketing products of algal origin as a functional food in the Coquimbo Region), sought to disseminate and promote the consumption of foods with algal origin in the population of the Coquimbo Region. This included making known their nutritional properties and the various recipes available for their preparation. The project also sought to develop new marketing formats for seaweed-based foods that are attractive to consumers in order to create new businesses for the Region’s seaweed producers and collectors. As a result, the cookbook “Comiendo Algas: Recetas de Cocina” (Eating Seaweed: Cooking Recipes) [67] was produced as well as “Manual de buenas prácticas: Cosecha y manejo de algas para el consumo alimenticio” (Good practice manual: Harvesting and handling of seaweed for food consumption) [68].

One of the latest projects that has focused on promoting the consumption of seaweeds is the “Nutrimar Biobio: Innovación en Alimentos Algales” (Nutrimar Biobio: Innovation in Algal Foods) project [69]. This project was conceived as an outreach project with the main goal being to implement an innovation platform for the diversification, valorization, and commercialization of edible seaweed resources from the Biobio Region, Chile. This included integrating seaweeds from this region into healthy food and nutraceutical products, and promoting the creation of new products, businesses, and markets at regional, national, and international levels. The project was based on the presence of bioactive compounds and the great availability of edible seaweed present on the Chilean coasts with a specialization focus on the Biobio Region. This included, but was not limited to, the seaweed species *Durvillaea antarctica* (“Cochayuyo”), *Ulva* sp. (“Sea Lettuce”), *Chondracanthus chamissoi* (“Sea Chicory”), *Pyropia* sp. and *Porphyra columbina* (“Luche”), and *Callophyllis variegata* (“Carola”). The “Nutrimar Biobio” project was extensive and contained a diversity of components. For example, the project developed prototypes of functional foods, such as snacks, cookies, and bread using *Ulva* sp. The initiative also allowed the development of nutraceutical product prototypes (soft capsules and soft-gel capsules) based on edible seaweeds *Durvillaea antarctica* and *Pyropia* sp. In addition, the project included outreach efforts to the general population through public participation in workshops. These workshops taught participants how to incorporate edible seaweed in several innovative recipes using *Durvillaea antarctica*, *Ulva* sp., *Chondracanthus chamissoi*, *Pyropia* sp., *Porphyra columbina*, and *Callophyllis variegata*. Other achievements obtained at the end of the project included outreach activities and training activities for organizations in the Biobio Region as well as high school students studying aquaculture and gastronomy; the creation of a recipe book containing 22 culinary recipes based on seaweed from the Biobio Region; the development of four cooking workshops with broad participation of seaweed collectors from the Biobio Region; seminars with algae marketing and exporting companies (both national and from the Biobio Region); the development of six seaweed nutrition sheets that include general information, recipes, and health benefits, among others characteristics; and seminars with the community focused on the identification, collection, management, cultivation, processing, and use of edible algae.

In summary, in the last decade, several projects have sought to encourage the Chilean population to consume local edible seaweed. All the projects examined have considered the different seaweed production stages. This includes the responsible management of natural seaweed beds, encouraging the cultivation of edible seaweed, training in the correct processing of seaweeds, and promoting seaweed consumption through the development of new products and foods, such as through culinary recipes.

## 6. Conclusions

In conclusion, recent research has demonstrated the economic and nutritional importance of edible seaweeds in many countries worldwide. Hence, the consumption of seaweeds and their products should be encouraged, but there is still exploration to do, especially in the field of the extraction of bioactive compounds that may have beneficial effects for human health. A challenge specifically for the food industry is to use the edible seaweeds or extracts for the development of innovative seaweed-based products. This includes decreasing the negative organoleptic impact that edible seaweed may have in foods. While seaweeds have many health benefits, they can also be a source of potentially toxic compounds, such as iAS. Fortunately, research has demonstrated that the levels of these compounds are below the maximum limit normally, particularly in the case of iAs. When these toxic compounds within seaweeds are not within permissible limits for safe consumption, alternative food products should be considered. Because of these potential risks, more research is needed to monitor the presence of these hazardous compounds, and then this information should be used to perform risk assessment analysis. In the case study of the county of Chile, several projects performed in the country in the last decade have demonstrated the need to promote the consumption of edible seaweeds, with very promising results. Given their overall successful results, the projects could be used as a model to promote the benefits of seaweeds in other countries. Overall, we emphasize the need to disseminate this knowledge throughout society in order to promote a social seaweed culture.

## 7. Future Directions

Due to the importance of edible seaweeds as both a future food source and supply of bioactive compounds with a wide variety of beneficial properties that could be used for human health, this review makes these main suggestions in order to promote the augmentation of these observed research gaps:

○ New methods of cultivation of edible algae under controlled conditions and away from areas with an anthropogenic presence to reduce exposure to potentially hazardous compounds for human and animal health.
○ Green chemistry for the extraction of bioactive compounds from seaweed.
○ The development of innovative seaweed-based food products.
○ Methods to encourage the consumption of seaweed in Western countries.
○ More research involving risk assessment based on seaweed consumption.
○ Normalizing the maximum limits of hazardous compounds in seaweeds and seaweed-based products.
○ Reporting the level or the presence of hazardous compounds in addition to the nutritional content of seaweed-based products so consumers know what they are consuming.
○ Monitoring should be extended to the “Emerging Pollutants”, which may also have detrimental effects on human and animal health.
○ Strengthen the dissemination of the benefits of seaweed consumption throughout society that also promotes a social algal culture and algal innovative ecosystem.

## Data Availability

Not applicable.
